# Regulating Precipitates by Simple Cold Deformations to Strengthen Mg Alloys: A Review

**DOI:** 10.3390/ma12162507

**Published:** 2019-08-07

**Authors:** Bo Song, Jia She, Ning Guo, Risheng Qiu, Hucheng Pan, Linjiang Chai, Changlin Yang, Shengfeng Guo, Renlong Xin

**Affiliations:** 1 School of Materials and Energy, Southwest University, Chongqing 400715, China; 2 College of Materials Science and Engineering, Chongqing University, Chongqing 400044, China; 3 Key Laboratory for Anisotropy and Texture of Materials (Ministry of Education), College of Materials Science and Engineering, Northeastern University, Shenyang 110819, China; 4 College of Materials Science and Engineering, Chongqing University of Technology, Chongqing 400054, China; 5 State Key Laboratory of Solidification Processing, Northwestern Polytechnical University, Xi’an 710072, China

**Keywords:** Mg alloys, precipitation, crystal defects, cold deformation, dislocations, twins, mechanical properties

## Abstract

Regulating precipitates is still an important issue in the development of high-strength Mg alloys, due to it determining the precipitation hardening effect. Cold deformation, as a simple and low-cost method, can remarkably influence the precipitate features. It is found that pre-cold deformation before aging can be utilized to enhance the precipitation hardening effect of Mg alloys. Moreover, post-deformation after aging could be an effective method to regulate precipitation orientation. In this review, recent research on the regulation of precipitation behavior by cold deformation in Mg-Al, Mg-Zn, and Mg-RE (RE: rare-earth elements) alloy systems was critically reviewed. The changes in precipitate features and mechanical properties of peak-aged Mg alloys via cold deformation were summarized. The corresponding strengthening mechanisms were also discussed. Finally, further research directions in this field were proposed.

## 1. Introduction

Precipitation hardening is the most remarkable hardening way and it plays a critical role in the development of high-strength Mg alloys [1,2]. The precipitation hardening effect is highly dependent on precipitate features (e.g., structure, size, shape, orientation, and distribution etc.) [3,4,5]. Thus, regulating precipitates is still one of the most concerned issues in Mg alloys researches.

Three aspects, i.e., major alloying elements, trace elements and heat treatment processes, generally control precipitation behavior. Major alloying elements, which can react with Mg to form precipitates with a higher volume fraction, are generally selected in order to achieve the purpose of aging strengthening. Moreover, the major alloying elements have an important influence on the structure and shape of precipitates. At present, the major alloying elements in Mg alloys mainly include Al, Zn, and RE [1]. For example, for Mg-Al alloy systems, the basal plate-shaped Mg_17_Al_12_ equilibrium phase with a complex body-centered cubic structure is reported as the precipitation strengthening phase [6]. For Mg-Zn alloy systems, the precipitation strengthening phase is a c-axis rod-shaped phase with a monoclinic crystal structure [7]. High-strength prismatic plate-shaped precipitate can be formed in the matrix of the Mg-RE alloy systems (e.g., typical Mg-Nd and Mg-Gd series alloys) [8,9].

In recent decades, the structure and strengthening mechanisms of these precipitates have been widely reported [6,7,8,9,10,11,12,13,14,15]. It is considered that it is very important for Mg alloys to enhance the precipitation hardening effect via promoting precipitation nucleation and regulating precipitation shape/orientation [3,4]. Microalloying is usually employed to change the precipitation behavior. Geng et al. [16] have reported that adding Cu, Ba, or Co to the Mg-Zn alloy can increase the eutectic temperature of the ternary alloy. It means that solution treatment can be performed at a higher temperature, which results in the increase in the number of precipitates during subsequent aging. Mendis et al. [17] have found that the addition of Ag in the Mg-5 wt.% Zn alloy can enhance the nucleation rate of the precipitates and make the rod-like precipitates more dispersed. Moreover, aging process can also influence the precipitation behavior. Traditional aging treatment generally adopts at a constant temperature. Recent studies have shown that multi-stage aging can effectively increase the nucleation rate of the precipitates [18,19].

Recently, enhancing the age-hardening effect by cold deformation has received increasing attention. Cold deformation before aging can introduce high density of crystal defects (e.g., dislocations, twins, stacking faults, et al.) [20,21,22,23,24,25,26]. Such defects can efficiency accelerate diffusion rate of atoms and offer more nucleation sites for precipitates during aging, thus improving age-hardening effect [21,22,23,24,25,26,27,28,29,30,31,32]. Clearly, the cold deformation method can be a significant supplement to the above-mentioned methods. Particularly, some simple cold deformation ways with low cost (e.g., uniaxial tension/compression [22,33,34,35,36], free-end torsion [27], low-strain rolling/forging [26,37,38,39,40], etc.) have exhibited a great application potential in the regulation of precipitates. As the most popular commercial Mg alloys, the precipitation behavior of Mg-Al, Mg-Zn, and Mg-RE alloy systems has been the most widely studied. In the previous studies, these alloy systems have also become the main research objects to study the influences of cold deformation on the precipitates. It is demonstrated that the cold deformation prior to aging in Mg alloys can effectively enhance the aging-hardening effect of various Mg alloys (including Mg-Al [27,28,29,30], Mg-Zn [31,32,33,34], and Mg-RE alloy systems [26,35,36,37,38]). Based on these alloy systems, the influences of cold deformation on precipitation features in Mg alloys were reviewed in detail in this review. The changes in mechanical properties and strengthening mechanisms of the Mg alloys that were processed by combining cold deformation with aging treatment were also discussed. Finally, further research directions on this field were proposed.

## 2. Influences of Pre-Cold Deformation on Aging Precipitation

### 2.1. Mg-Al Alloy Systems

**Figure 1:**
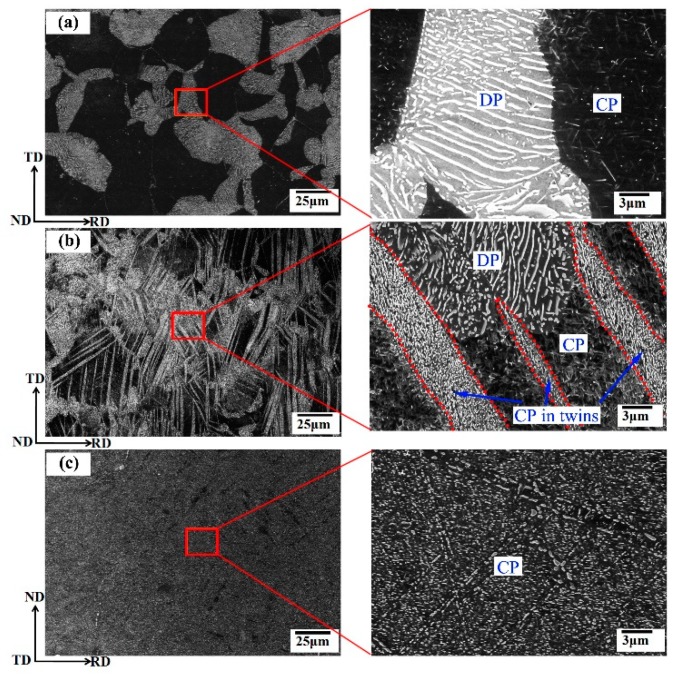
Scanning electron microscope (SEM) micrographs of rolled AZ80 alloys (**a**) direct aged, (**b**) compressed 3.5% along TD and then aged and (**c**) compressed 10% along TD and then aged. Red dotted lines in (**b**) outline twin boundaries. The micrographs at the right-side were taken at higher magnification from the corresponding marked areas [28].

Mg-Al alloy systems are the most widely used of Mg alloys. The addition Al element not only improves castability, but it also enhances strength and ductility of Mg alloys at room temperature. Generally, Mg_17_Al_12_ phase is main precipitates in the Mg-Al alloy systems with high Al content (e.g., AZ91, AZ80, and AZ61, etc.). It usually has a plate-like morphology on the basal plane with a Burgers orientation relationship with the matrix (i.e., (0001)_α_//(011)_β_ and (2-11-0)_α_//(11-1)_β_) [6]. The Mg_17_Al_12_ precipitate has two types of distribution states: coarse discontinuous precipitates (DP) and fine continuous precipitates (CP) [28,41], as shown in Figure 1a. The two types of precipitates usually simultaneously occur and compete each other. Fine continuous precipitate is generally believed to provide the major age-hardening effect in Mg-Al based alloys [28]. Thus, promoting continuous precipitates and regulating their size and density are key in raising the age-hardening effect of the Mg-Al alloy systems. It has been reported that the precipitation type is dependent on the dominated diffusion mechanism during aging [41]. When the grain boundary diffusion process dominates, the discontinuous precipitates are favored, and continuous precipitates are formed when the volume diffusion becomes faster. Cold deformation can generate a high density of crystal defects within grains, which can promote the volume diffusion during aging. Thus, cold deformation could remarkably influence the precipitation behavior of the Mg-Al alloy systems.

Yang et al. [30] have confirmed that cold rolling with 10% reduction prior to aging can promote precipitation of fine particles during aging (at 300 °C/1 h) of the as-cast AZ80 alloy. In their study, cold rolling can also generate some deformation twins, except dislocations. It is found that deformation twins via cold rolling can promote continuous precipitation more effectively when compared with dislocations [30]. Thus, it is inferred that pre-inducing a large number of deformation twins may effectively promote continuous precipitation during aging. When compared to as-cast alloys, wrought Mg alloys can generate profuse {10–12} twins by deformation along special directions [42]. Zhang et al. [43] have fabricated an extruded AZ61 rod containing profuse {10–12} twin lamellae by compression along extrusion direction (to a plastic strain of ~3%). The preferential nucleation of precipitates in the twin boundaries and within twins has also been observed in pre-twinned AZ61 alloy during aging at 180 °C. Recently, Wang et al. [28] compared different precipitation behavior between the twinned region and untwinned matrix in a rolled AZ80 plate, as shown in Figure 1. It is found that only continuous precipitates can be observed within {10–12} twins during aging at 180 °C (see Figure 1b). The untwinned matrix exhibit similar precipitation features with aged sample without pre-twinning (see Figure 1a,b). Wang et al. [28] fabricated a complete twinned AZ80 plate (twinned area fraction of ~94%) by compression to a strain of 10% along TD to achieve more uniform continuous precipitation in the entire matrix (see Figure 1c).

**Figure 2:**
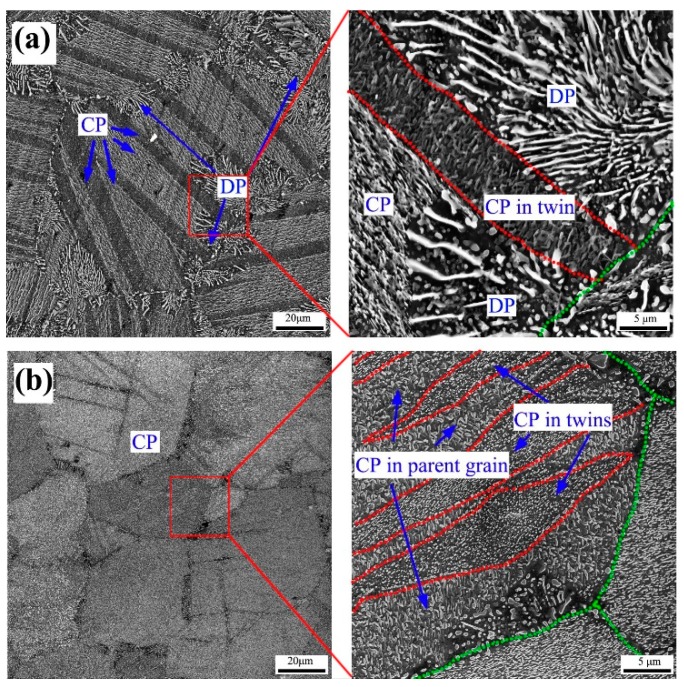
SEM micrographs extruded AZ91 rods which subjected torsion and subsequent aging (**a**) center position and (**b**) edge position. Green dotted lines and red dotted lines outline the grain boundaries and twin boundaries, respectively. The micrographs at the right-side were taken at higher magnification from the corresponding marked areas [27].

Previous studies have shown that twin structures can be the favorable nucleation sites only for the continuous precipitates during aging. Additionally, the precipitation behavior within {10–12} twins is independent on pre-twinning strain amount before aging [27,28]. In fact, slipping deformation can also increase the continuous precipitation in untwinned region of the Mg-Al alloy systems. Recently, Song et al. [27] found that slipping deformation via free-end torsion can also promote continuous precipitation in an extruded AZ91 rod during aging at 180 °C. With increasing shear strain, the amount of continuous precipitation in untwinned region gradually increases, as shown in Figure 2. Clearly, unlike the {10–12} twins, the precipitation behavior of untwinned region largely depends on the strain amount of slipping deformation.

It has been confirmed that cold deformation can promote continuous precipitation of Mg-Al alloy systems. In general, it is attributed to the result that the crystal defects (dislocations, stacking faults, etc.) via cold deformation promote volume diffusion [41]. However, it is still unclear why twinning deformation and slipping deformation exhibit different influences on precipitation behavior. Some studies on Mg and its alloys found that a large number of non-basal dislocations and basal stacking faults can be formed inside {10–12} twins that are not easily activated in parent grains [44,45,46]. The occurrence of such crystal defects inside twins can be attributed to interactions between the matrix dislocations and the twin boundaries. It is considered that these special crystal defects within twins could be the favorable nucleation site for CP. However, this speculation and its micro-mechanism require more work to interpret. Moreover, most of the previous work on the precipitation features in Mg-Al alloy systems is based on the scanning electron microscope (SEM) technique. Thus, the influence of cold deformation on size and shape of precipitates in the Mg-Al alloy systems lacks effective evidence. More detailed characterization by multiple characterization techniques (e.g., transmission electron microscope (TEM)) is very necessary. At latest, Liu et al. [47] reported that pre-compression could dramatically reduce the aspect ratio of the Mg_17_Al_12_ particles and affect the orientation of Mg_17_Al_12_ phase. Gu et al. [48] found that the precipitates on the twin boundaries are rod-like, while others are lath shaped.

### 2.2. Mg-Zn Alloy Systems

Mg-Zn alloy systems are also an important commercial Mg alloy. The Mg-Zn binary system is usually used to investigate the precipitation phase structure [12,49]. Based on Mg-Zn binary alloys, some new alloys have been developed, e.g., Mg-Zn-Zr [33], Mg-Zn-Mn [50], Mg-Zn-Ca [51], and Mg-Zn-Y [52], etc. For Mg-Zn alloy systems, MgZn_2_ is the main precipitates and can form two main precipitation shapes during aging, i.e., rod-like β_1_′ phases along c-axis and disc-like β_2_′ phases lying flat on basal plane [12].

**Figure 3:**
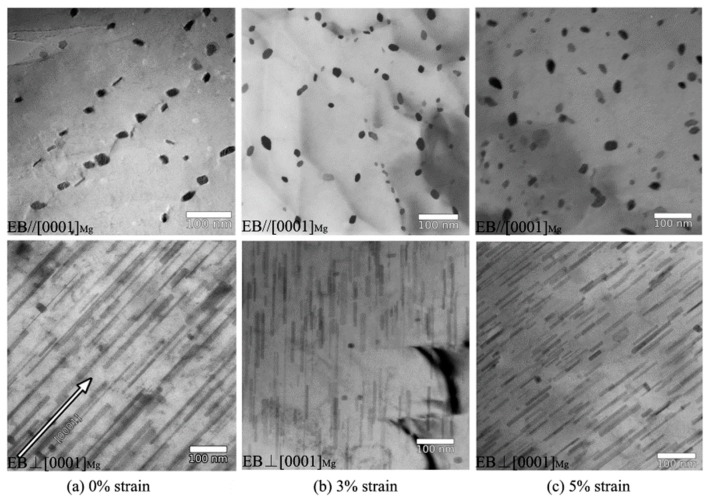
Transmission electron microscope (TEM) of β′_1_ precipitates in the optimum hardness conditions as a function of pre-tension deformation in an extruded binary Mg-3.0 at.% Zn alloy. (**a**) 0% strain, (**b**) 3% strain, and (**c**) 5% strain. EB is electron beam direction [55].

In the Mg-Zn binary system, the rod-like β_1_′ phase has a main contribution on peak aging (i.e., aging on maximum hardness) and it largely influences yield strength and anisotropy [4,53]. It is found that pre-cold deformation can accelerate precipitation and enhance the age-hardening effect of the Mg-Zn binary alloys, as reported by Pan et al. [31] and Clark [54]. For Mg-Zn binary alloys, the dislocations and twins show a distinct effect on precipitation. Clark et al. [54] used cold rolling to treat an as-cast Mg-5 wt.% Zn alloy. It is found that dislocations can refine precipitates, but the deformation twins result in coarsening of the β_1_′ particles. It indicates that deformation twins may be detrimental to age-hardening response of the Mg-Zn binary alloys. It is known that the tension perpendicular to c-axis (i.e., tension along the extrusion direction of extruded Mg alloy rods) can suppress {10–12} twinning [42]. Rosalie et al. [55] applied pre-tension (3% and 5%) along the extrusion direction (ED) to an extruded Mg-3.0 at.% Zn to induce profuse dislocations, instead of twins. It was found that high density of dislocations via slipping deformation can effectively refine the size of β_1_′ phases, as shown in Figure 3. Shi et al. [50] used pre-tension along ED to enhance the age-hardening effect of an extruded Mg−6%Zn−1%Mn alloy. It indicates that dislocations can be the preferred nucleation sites for the β_1_′ phase. The number density of β_1_′ rods also increases with the rise in the pre-deformation level.

**Figure 4:**
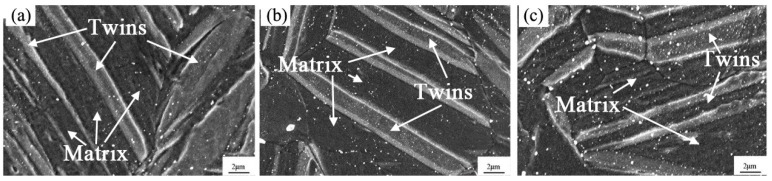
SEM micrographs of the distribution of precipitates in the pre-twinned ZK60 samples aged at 180 °C for (**a**) 3 h; (**b**) 10 h; and, (**c**) 50 h [33].

ZK60 is a typical commercial Mg-Zn-Zr alloy and it possesses relatively high yield strength and good plasticity [56]. Disk-like β′_2_ precipitates contributed to the hardening at the beginning of aging, and their coarsening is responsible for over-aging of ZK60 alloys [57,58]. Similar to Mg-Zn binary alloys, cold deformation (cold rolling, cold compression etc.) can also promote the precipitation of MgZn_2_ and enhance the age-hardening effect of ZK60 alloys [32,59]. The difference is that deformation twins can refine the precipitates of ZK60 alloys [33,34]. Song et al. [34] found that pre-induced twin boundaries in rolled ZK60 plate can be the favorable sites for the nucleation of MgZn_2_ precipitates after aging at 175 °C for 10 h. Chen et al. [33] have systematically investigated the influence of aging time (at 180 °C) on the precipitation behavior of pre-twinned ZK60 plate that is fabricated by pre-compressing 3% along TD. It is found that pre-twinning has little influence on the precipitation type. However, the number density of precipitates in the twinned region is still higher than that in the un-twinned region, as shown in Figure 4. Thus, it is considered that the pre-inducing {10–12} twins can enhance the age-hardening effect in ZK60 alloys.

**Figure 5:**
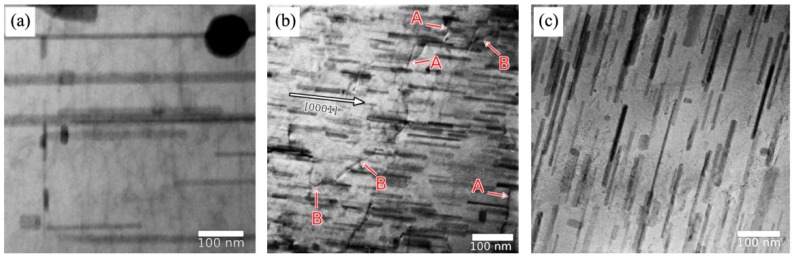
Transmission electron micrographs of β′_1_ rod-shaped precipitates in the peak aged condition of an extruded Mg-Zn-Y alloy, showing β′_1_ precipitates viewed normal to the hexagonal axis of magnesium for (**a**) 0%, (**b**) 3%, and (**c**) 5% pre-aging strain. Dislocations are visible in the foil given 3% strain (**b**). Segments of dislocations that are parallel to the basal plane are labelled “A” and segments of dislocations that lie at an angle to the basal plane are labelled “B” [52].

Recently, Mg-Zn-RE alloys have received more attention, among which Mg-Zn-Y alloys is typical one of such alloys [60,61,62,63]. Y additions can delay over-aging in Mg–Zn alloys [52]. Moreover, Mg-Zn-Y alloys with Zn:Y ratios of ~6:1 can precipitate a quasicrystalline phase (i-phase, Mg_3_Zn_6_Y) [64]. The formation of the i-phase could reduce the precipitation of β_1_′ phase, resulting in low aging hardening response. Rosalie et al. [52] have found that dislocations via pre-tension along the ED can re-partition Zn to β_1_′ precipitates in an aged Mg-Zn-Y alloy (Mg-3.0 at.% Zn-0.5 at.% Y). Additionally, they can also provide heterogeneous nucleation sites for the β_1_′ precipitates and increase the volume fraction of the β_1_′ precipitates, as shown in Figure 5. Moreover, deformation twins can also be used to refine the β_1_′ precipitates of Mg-Zn-Y alloys. Ye et al. [65] used pre-compression to introduce {10–12} twins, {10–11}–{10–12} double twins, and dislocations in Mg-9.02Zn-1.68Y alloy. After pre-compression at a low-strain (3–15%), the density of β′_1_ precipitate gradually increases and the size decreases with increasing the pre-strain. Additionally, it is also found that the size of precipitates within twins is smaller than that within the matrix, and the precipitates in the twin boundaries can be identified as the granular i-phase.

According to above, the relationship between precipitation and dislocations during aging can also be observed, and the initial dislocations could also influence the partition of alloying elements during aging precipitation. These results provide an important reference for regulating precipitation behavior. Moreover, it is found that deformation twins exhibit different influences on precipitation behavior between Mg-Zn binary and ternary alloys. For Mg-Zn binary, initial deformation twins lead to a coarsening of precipitates. However, deformation twins can refine precipitates for some Mg-Zn ternary alloys (Mg-Zn-Zr, Mg-Zn-Y etc.). The alloying element seems to affect the role of deformation twins. However, the essential reason remains unclear. Mg-Zn alloy systems contain various shapes of precipitates. Controlling precipitate shape can remarkably influence the strength and anisotropy of Mg-Zn alloy systems [66]. However, the effect of cold deformation on the precipitate shape has not received enough attention.

### 2.3. Mg-RE Alloy Systems

The addition of rare-earth elements has great impact on deformation mechanisms and recrystallization behavior of Mg alloys [67,68,69]. Moreover, basing on Mg-RE alloy systems, some high-strength Mg alloys (e.g., Mg-Y-Nd, Mg-Gd-Nd, Mg-Dy-Nd, and Mg-Gd-Y, etc.) are also developed and their high strength is achieved essentially via precipitation hardening [70,71,72]. Mg- RE alloy systems usually have a precipitation sequence of SS (solid solution)—β″ → β′ → β_1_ → β during static precipitation [2,73]. The formation of the coherent β″ phase leads to a slight increase in hardness, but the peak hardening is caused by precipitation of dispersed fine semi-coherent β′ particles. The particles of the stable β_1_ and β phase are completely incoherent with the matrix and they only cause a negligible hardening [74].

It has been reported that cold deformation can accelerate precipitation and enhance the precipitation hardening response of Mg-RE alloy systems [75]. Moreover, the influence of cold deformation on various types of precipitates via subsequent aging has also been reported. Zheng et al. [36] found that high density of dislocations and twins that were introduced by pre-stretching (5% and 10%) can facilitate the rapid growth of β_1_ in a Mg-11Gd-2Nd-0.5Zr (wt.%). However, such pre-introduced defects do not increase the number density of β″/β′ precipitates. Čížek et al. [76] examined the effect of dislocation density on the precipitation process in Mg-15 wt.% Gd alloy by cold rolling. It is found that precipitation of the β″ phase is insensitive to dislocation density, while dislocations promote nucleation of the β’ precipitates. Zhao et al. [35] used pre-compression (2–10%) to treat an as-solid solution Mg-2.7Nd-0.4Zn-0.5Zr alloy and found that pre-compression increased the density of β″ precipitates. Kang et al. [24] found that multi-directional forging can refine the β″ and β′ phases and accelerate the formation of β_1_ phase in a WE43 alloy (Mg-4.38Y-2.72Nd-1.10Gd-0.56Zr (wt.%)). Li et al. [38] reported that cold rolling can lead to a higher density of β′ precipitates in the extruded Mg-14Gd-0.5Zr matrix during subsequent aging. Li et al. [26] also used cold rolling to refine the β″ precipitates and increase its density in an as-cast Mg-2.1Gd-1.7Ho-1.4Y-1.3Nd-0.9Er-0.5Zn-0.5Zr alloys.

**Figure 6:**
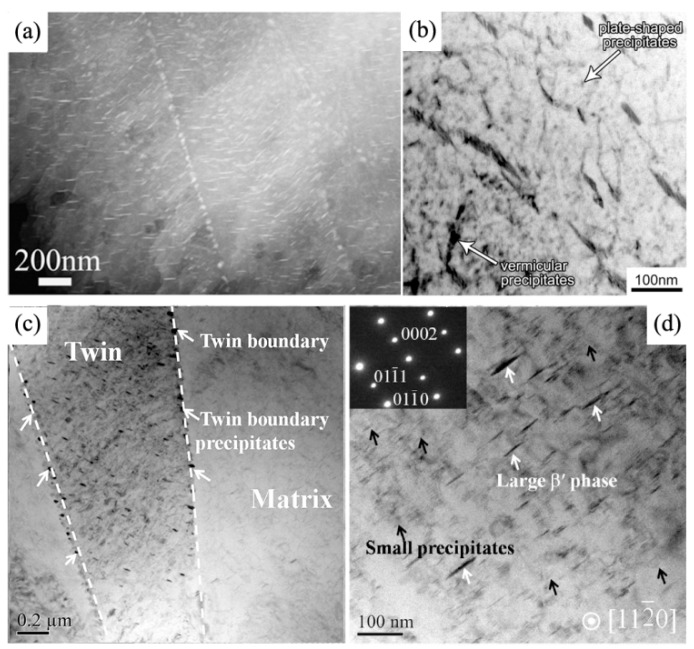
TEM micrographs of various samples (**a**) extruded Mg-4Sm alloy subjected 20% cold rolling and aging [37], (**b**) the Mg−2.7Nd−0.4Zn−0.5Zr alloy with a pre-compressive strain of 8% and subsequently aged at 200 °C or 8 h [35], (**c**,**d**) are the twin boundary precipitates and matrix interior precipitates in the pre-deformed WE43 alloy, respectively [24].

Cold deformation can also influence the distribution and shape of precipitates, as shown in Figure 6. Li et al. [37] found that cold deformation can induce more uniform precipitation in the matrix of the Mg-4Sm alloy. It is found that cold rolling remarkably suppresses the formation of precipitate-free zone in extruded Mg-4Sm alloy during subsequent aging. Moreover, some bulk Mg_3_Sm particles can be observed in twin boundaries (see Figure 6a). Zhao et al. [35] found that the aging induced not only plate-shaped β″ precipitates, but also vermicular β″ precipitates in the pre-compressed Mg−2.7Nd−0.4Zn−0.5Zr alloy (see Figure 6b). Kang et al. [24] reported that a few spheroid–shaped precipitates and relatively large β′ precipitates can be dynamically formed at twin boundaries and within matrix, respectively, during the pre-forging of WE43 alloy, (see Figure 6c,d).

It has shown that cold-deformation does not change the precipitation sequence, but it accelerates the precipitation and facilitates the growth of β_1_. It indicates that cold deformation can shorten the time of over-aging. It is expected that β″/β’ precipitates can be regulated by pre-cold deformation to enhance the aging-hardening response. As reviewed above, there are some different results on the influence of cold deformation on β″ and β’ precipitates. The reason needs to be revealed in the further work. It is also shown that cold deformation can also promote the uniform precipitation, which is good for strength and toughness. Moreover, it is also reported that a small amount of new precipitates can be formed by pre-cold deformation. It is necessary to evaluate their effect on mechanical properties.

The above has reviewed the influence of cold deformation on precipitation behavior of Mg-Al, Mg-Zn, and Mg-RE alloy systems. Microalloying can influence microstructure evolution and precipitation behavior during deformation and heat treatment [1,16,17,68]. Thus, it is usually used to further improve the mechanical properties of existing Mg alloys. Based on this, some typical Mg alloys (e.g., Mg-Al-Si, Mg-Al-RE, Mg-Al-Mn, Mg-Zn-Ca, Mg-Zn-RE, and Mg-RE-Ag, etc.) have been widely reported [1,71]. Moreover, some new Mg alloy systems (Mg-Ti, Mg-Ca, and Mg-Sn based alloys, etc.) have also been developed [1]. It is considered that pre-cold deformation can also effectively enhance the age-hardening effect of these Mg alloys. However, the influence of cold deformation on the precipitation behavior of these alloy systems is still lacking systematic research. Recently, Hu et al. [22] and Fang et al. [77] used pre-cold deformation (pre-tension and pre-cold rolling, respectively) to refine the Mg_2_Sn phase and increase the number density of precipitates in Mg-Sn based alloys.

## 3. Deformation Induced Transition in Precipitate Orientation/Shape

In addition to size and density of precipitates, the shape and orientation of precipitates can also affect the mechanical properties of Mg alloys. It has been reported that the precipitate orientation (i.e., the orientation relationship between precipitated phase and the matrix) has important influence on the strength and anisotropy of Mg alloys [3,4]. As reviewed above, the precipitate shape in Mg alloys is very rich, e.g., basal plate-shaped precipitates in Mg-Al, prismatic rod-shaped precipitates in Mg-Zn alloy, and prismatic plate-shaped precipitates in Mg-RE alloy, etc. Clearly, for a specific Mg alloy, the shape and orientation of precipitates are generally definite. It is reported that the shape of a precipitate is determined by the interplay between interfacial energy and elastic strain energy [73]. Thus, the change in shape and orientation of precipitates is very difficult by traditional aging.

**Figure 7:**
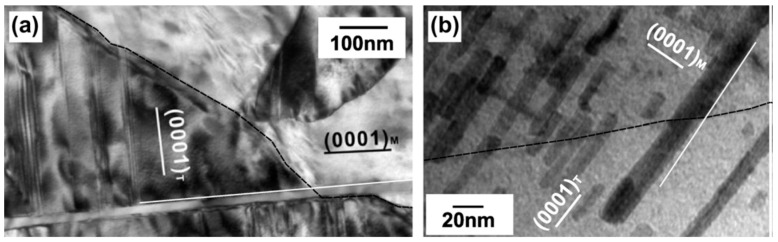
Interaction of a {10–12} twin and particles in peak-aged material (**a**) basal plate-shaped precipitate in AZ91 alloy. (**b**) c-axis rod-shaped precipitate in Mg-5%Zn alloy. The twin boundary has been highlighted with a dashed line for clarity [4].

**Figure 8:**
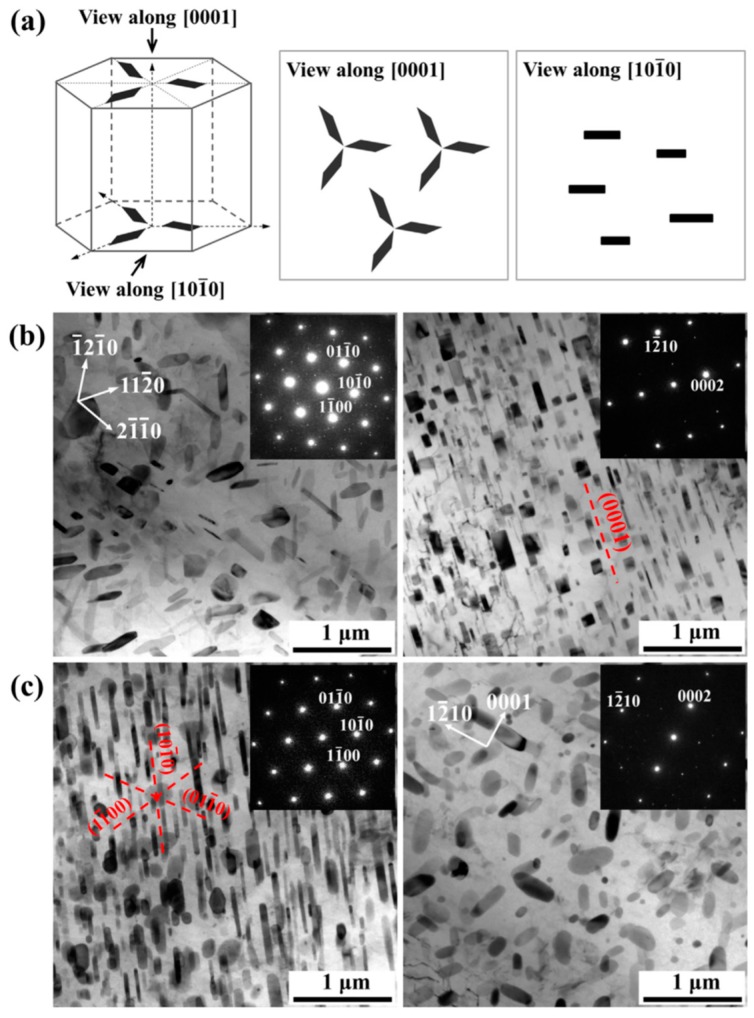
(**a**) Diagrams illustrating the morphologic characteristics of Mg_17_Al_12_ precipitates viewed from [0001]_α_ and ${\left[10\bar{1}0\right]}_{\alpha }$ TEM micrographs of various samples subjected to (**b**) twinning deformation- aging and (**c**) twining deformation-aging-detwinning [11].

Besides alloying, stress-aging is also an alternative method for changing the precipitate shape. Peng et al. [78] reported that aged at 190 °C under an applied stress of 30 MPa can change the precipitates shape from prismatic plate to spherical precipitate in a Mg-5 wt.%Sc alloy. Zhao et al. [79] also used a super high-pressure aging (6 GPa at 300 °C) to achieve a spherical precipitate in a Mg-10Y alloy. Depending on previous reports, it seems that cold deformation before aging cannot largely affect the shape and orientation of precipitation phase via subsequent aging in Mg alloys. However, the post-cold deformation after aging is considered to be a potential method for regulating the precipitation orientation. Plastic deformation can cause rotation of the lattice, but the precipitates may not rotate with the matrix [80,81,82]. Thus, for the Mg alloys with initial precipitates, subsequent cold deformation could change the orientation relationship between the precipitates and matrix. It is dependent on the interaction between slip/twinning and precipitates. In fact, the interaction between {10–12} twins and precipitates has been widely reported in Mg-Zn, Mg-Al and Mg-RE alloy systems [4,81,82]. Figure 7 shows the orientation relationship between the precipitates and matrix (parent grain and {10–12} twin). It showed that twinning/detwinning arouse ~86.3° rotation of the matrix, however only arouse a small rotation of the precipitate in twin (<10°) [4]. Thus, twinning-detwinning after aging can achieve a large re-orientation of precipitates. Recently, Liu and Xin et al. [11] used coupling twinning, aging, and detwinning method to regulate the precipitates orientation of rolled AZ80 alloy from basal precipitates to prismatic precipitates, as shown in Figure 8. Clearly, the distribution of precipitates with different orientation can also be tailored by controlling twinning process. In the further work, the precipitates orientation transition mechanism during twinning-detwinning needs to be revealed. Systematic work regarding control in distribution of precipitates with different orientation by twinning-detwinning could be a key problem in this field.

## 4. Strengthening Mechanism via Combined Use of Cold Deformation and Aging

**Figure 9:**
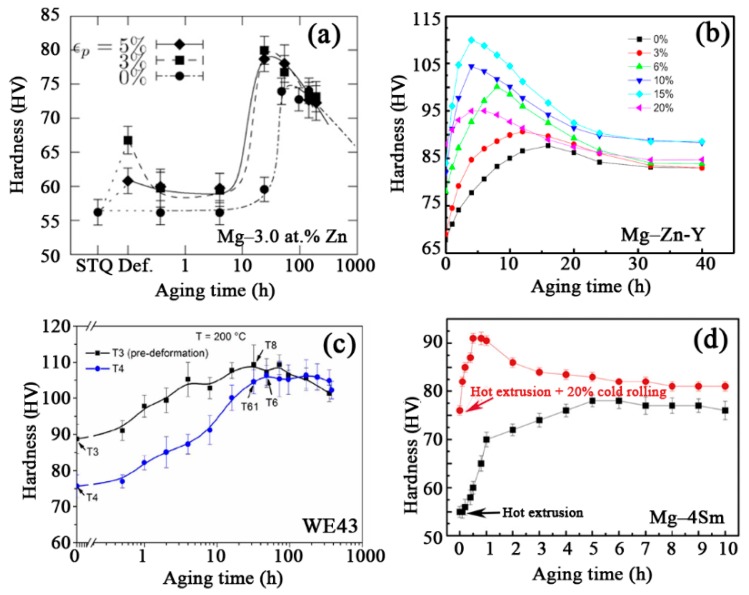
The aging hardness curves of pre-strained alloys. (**a**) Extruded Mg-3.0at.%Zn alloy (pre-tensile strain along extrusion direction (ED)) [55], (**b**) as-cast Mg-7.68Zn-1.68Y alloy (pre-compression) [65], (**c**) as-cast WE43 alloy (pre-cold forging) [24], and (**d**) extruded Mg-4Sm alloy (pre-cold rolling) [37].

Micro-hardness test is usually used to evaluate the age-hardening effect. Figure 9 shows some typical age-hardening curves. In general, cold deformation shortens peak aging time and increases the hardness of peak aging. It can be mainly attributed to crystal defects being introduced by cold deformation accelerating the diffusion rate of atoms and promoting the nucleation of precipitates. Clearly, pre-cold deformation not only enhances age-hardening effect, but it also reduces cost and energy consumption. Usually, tension and compression tests are carried out to investigate the strength, anisotropy, and ductility. Table 1 summarized the tensile properties of various Mg alloys underwent pre-cold deformation and subsequent peak aging. Similar to micro-hardness, the tension yield strength of Mg alloys can be remarkably enhanced by the combined use of pre-deformation and aging treatment. Moreover, for Mg-Al and Mg-Zn alloy systems, pre-cold deformation enhances subsequent age-hardening effect without sacrificing the ductility.

The stress increment via precipitation hardening can be evaluated by the Orowan mechanism. According to the Orowan equation ($\Delta \sigma_{Orowan}=\frac{Gb}{2\pi \sqrt{1-\nu }}\frac{1}{\lambda }\ln\frac{d_{p}}{r_{0}}$, where *G* is the shear modulus, *b* the Burgers vector of the gliding dislocations, *ν* the Poisson ratio, *λ* the effective interparticle spacing on the slip plane, *dp* the mean planar diameter of the particles on the slip plane, and *r_0_* the dislocation core radius.), the precipitate features (density, amount, size, shape, and distribution, etc.) can remarkably influence the precipitation hardening effect [3,4]. As reviewed in Section 2, crystal defects via pre-deformation can provide additional nucleation sites for precipitation during subsequent aging. Thus, pre-cold deformation can remarkably increase their number density, which results in an enhancement of the age-hardening effect. Moreover, it has also been confirmed that particles with different shape and habit will strengthen different deformation modes to different extents [3,4]. A typical example, basal plate-like precipitation in the Mg-Al alloy systems has a stronger hardening effect on {10–12} twinning than on the prismatic slip. The deformation that was induced by twinning-detwinning after aging can largely change the precipitate orientation in specific Mg alloys [4,11,81,82], as reviewed in Section 3. Liu and Xin et al. [11] also confirmed that the change in precipitates orientation via detwinning (from basal-plate precipitates to prismatic-plate precipitates) can enhance the precipitation hardening effect on the yield strength dominated by slipping in rolled AZ80 alloy. Thus, it is expected that the transition of precipitate orientation induced by deformation can further tailor the mechanical properties and expand the application of existing Mg alloys.

In fact, cold deformation not only influences the amount and density of precipitates, but also their size and distribution. As listed in Table 2, pre-deformation can refine precipitates and could change the aspect ratio of precipitates. Nie et al. [2] found that the aspect ratio can also remarkably influence the hardening effect. It is pointed out that the prismatic plates with a large aspect ratio can more effectively block dislocations and twin motion. Therefore, the effect of pre-deformation on the aspect ratio of precipitates should be concerned. Moreover, the influence of distribution of precipitates on mechanical properties has not received enough attention. Deformed microstructure features determine the distribution of precipitates. It can be used to promote the uniform precipitation, as reported by Li et al [37]. Moreover, it can also be used to generate non-homogeneous distribution of precipitates, as reported by Song et al. [27], Wang et al. [29] and Liu et al [11]. For example, torsion deformation induces gradient strain in rods, resulting in macroscopic non-uniform distribution of precipitation behavior [27]. Transition of precipitation behavior relating to deformation twins can induce microscopic non-uniform distribution of precipitates [11,29]. Recently, the heterogeneous structure (e.g. gradient microstructure, multilayers, bi-model structure etc.) has received more and more attention, because it can balance strength and toughness compared with uniform microstructure [83,84,85,86,87,88]. Thus, design of precipitation distribution by cold deformation and its influence on mechanical properties could be an important research direction in the further study.

The increment of yield strength via the combined use of cold deformation and aging can be mainly attributed to increased precipitation strengthening. Moreover, cold deformation can also induce a large number of crystal defects (including dislocations, twins, and stacking faults, etc.) and arouse textural change [89,90,91,92]. In general, aging treatment is usually carried out at lower temperatures (<250 °C). Thus, aging treatment usually only arouses static recovery, instead of static recrystallization, in pre-deformed Mg alloys. Deformation twins, deformation texture, and part of dislocations via pre-cold deformation can usually be remained after aging [27,28,29,30,31,32,33,34,35,36,37,38]. These microstructural changes can also generate a hardening or softening effect in yield strength. Therefore, the influence of the deformed microstructure on strength should also be taken into account when evaluating the effect of cold deformation on the age-hardening effect.

Dislocations can strengthen the alloy by interacting with themselves and impeding their own movements. Residual dislocations can generate a dislocation-strengthening effect, which is dependent on the dislocation density. The increment in yield strength via residual dislocations can be evaluated by the Bailey–Hirsch equation: $\Delta Re_{dislocations}=M\alpha Gb\rho^{\frac{1}{2}}$, where *M* is the Taylor factor, α is a dislocation-dislocation interaction constant, *G* is the shear modulus, *b* is the Burger vector and ρ is the dislocation density [93]. {10–12} twinning can be easy to induce in Mg alloys (especially for Mg alloys free RE) during cold deformation. Twin boundary is a special grain boundary. Twins lamellae can also subdivide grains and act as useful barriers to dislocation slip [94]. Similar to grain boundaries, the twin boundary strengthening can also be established by a Hall–Petch-type relationship as: $\Delta Re_{TB}=V_{f}K^{TB}\lambda_{TB}^{-1/2}$, where V_f_ is the twin volume fraction, *K^TB^* is a parameter that describes the relative strengthening contributions of twin boundaries, and *λ_TB_* is the average twin-boundary spacing [95].

Moreover, stacking fault could also be formed in Mg and its alloys under particular conditions [46,61,90,96]. The formed stacking faults can hinder the dislocation movement, which results in an increase of strength [97,98]. It has been reported that the composition and loading rate/orientation can affect the formation of stacking faults in Mg alloys. Previous has showed that the formation of stacking faults is usually related to the incoherent twin boundary migration [99]. Thus, the stacking faults are not easily produced in the matrix, and they are usually observed within deformation twins in Mg and its alloys [46,99,100,101]. Recently, Li et al. [26] found basal plane stacking faults can be widely formed in Mg matrix with multi-RE alloying (Mg-2.1Gd-1.7Ho-1.4Y-1.3Nd-0.9Er-0.5Zn-0.5Zr (wt.%)) after cold-deformation. In their study, the contribution of stacking faults to strength was calculated by a reported equation in Ref. [97,98]. The stacking faults with a volume fraction of 0.2% contribute a yield strength increment of 33.9 MPa.

For Mg alloys, cold deformation can also arouse textural change and arouse texture hardening/softening [83,102]. Pre-deformation via slip deformation at a low-strain generally exhibits little influence on texture. However, {10–12} twins can be induced at low strain when deformation occurs along special orientation and generates a large re-orientation [94]. Moreover, some simple deformation modes (e.g., cold rolling, torsion deformation, etc.) can achieve large strain and remarkably change texture by slipping deformation [26,83]. Li et al. [38] reported that pre-cold rolling not only refined precipitates of an extruded Mg-RE alloy, but it also enhanced basal texture to generate a texture hardening effect on subsequent tension. Song et al. [103] found that unidirectional torsion deformation enhanced the age-hardening effect of an extruded AZ91 rod, but generated a texture softening effect on tensile strength. Thus, the influence of textural change via cold deformation (even low strain deformation) on strength should be taken seriously.

## 5. Conclusions and Outlooks

1. Pre-cold deformation before aging can accelerate precipitation and enhance the age-hardening effect. It has been widely used to enhance the strength of peak-aged Mg alloys. It is generally believed that this is due to crystal defects via cold deformation providing heterogeneous nucleation sites. Although some researchers have found that the precipitates favorably nucleated on twin boundaries and dislocations, the micro-mechanism has been lacking in-depth systematic investigation. The relationship between the type of crystal defects and the features of precipitates needs to be built.
2. As reviewed above, dislocations and deformation twins usually exhibit different influences on the precipitation behavior in Mg alloys, especially in Mg-Al and Mg-Zn alloy systems. It could be related to the different crystal defects evolution in parent grain and twins, as discussed in Section 2.1. Moreover, microalloying could change the influence of dislocations or twins on precipitation behavior, as discussed in Mg-Zn and Mg-RE alloy systems. For these phenomena, micro-mechanism is unclear and it needs to be further revealed.
3. Post-cold deformation after aging can be an optional method for regulating precipitate orientation. It has been confirmed that twinning-detwinning can remarkably change the orientation relationship between precipitates and Mg matrix. As a simple and low-cost method, it is considered that it has large potential as a regulation technology of precipitate orientation. Currently, this method is less useful in improving the strength/toughness and anisotropy of magnesium alloys. The study on the transition of precipitation orientation and its influence on mechanical properties will be key in revealing the effect of this method.
4. Cold deformation can promote uniform precipitation and eliminate the precipitate-free zone in Mg-RE alloy systems. Moreover, cold deformation can also induce non-uniform distribution of precipitates. It is closely related with features and distributions of crystal defects, which are controlled by the strain state. It is expected that the optimized heterogeneous precipitation could exhibit better comprehensive properties. Thus, it is necessary to develop Mg alloys with heterogeneous precipitation via cold deformation, and revealed the relationship between heterogeneous precipitation and mechanical properties.
5. Cold deformation can influence precipitate features, which resulted in a change in the precipitation hardening effect. It should be also pointed out that cold deformation could also generate deformed microstructure (e.g., dislocations, twins, stacking faults, deformation texture, etc.), which could arouse an additional hardening/softening effect. Multiple structure control, including precipitates and deformed microstructure, should be taken into account to evaluate the change and optimization in mechanical properties.

## Figures and Tables

**Table 1:** Mechanical properties of various Mg alloys subjected to different treatments. The sample sizes for mechanical testing can be found in the corresponding references.

Materials	Deformation before Aging	Pre-Strain Amount	Loading Direction	*R*_p0.2_ (Mpa)	*R*_p0.2_ (Mpa)	Elong (%)	Elong (%)
				Before Aging	After Aging	Before Aging	After Aging
As-cast	Tension	0%	Tension	134	222	21	2.5
Mg–11Gd–2Nd–0.5Zr [36]		5%			276		2
		10%			298		1.4
As-cast	Forging (Multi-axial)	0%	Tension	155	204	6.9	1.1
WE43 [24]		15%			272		0.7
Extruded	Rolling (ED)	0%	Tension (ED)	90	155	10	6
Mg–4Sm [37]		20%					2
Extruded	Rolling (ED)	0%	Tension (ED)	190	305275	19.5	7.0
Mg-14Gd-0.5Zr [38]		10%		255	375	12	3.0
		19%		295	420	4.5	2.3
		27%		305	445	3.5	2.0
As-cast	Rolling	0%	Tension (RD)	132	147	14.8	8.9
Mg-2.1Gd-1.7Ho-1.4Y-1.3Nd-0.9Er-0.5Zn-0.5Zr [26]		12%		224	259	6.5	5.4
Rolled	Compression (TD)	0%	Tension (RD)	171	202	12.6	7.2
AZ80 [28]		10%			283		9.1
Extruded	Tension (ED)	0%	Tension (ED)	143	273	0.25	16
Mg–3Zn [55]		3%			305		15
		5%			309		15
Rolled	Compression (ED)	0%	Tension (RD)	185	223	18.4	14.2
ZK60 [34]		3%		217	258	16.6	16.6
Extruded	Tension (ED)	0%	Tension (ED)	204	320	14	6
ZM61 [50]		5%			347		6
		10%			356		4
Extruded	Tension (ED)	0%	Tension (ED)	150	217	-	-
Mg-3Zn-0.5Y [52]		3%			281		
		5%			287		
Rolled	Tension (RD)	0%	Tension (RD)	185	196	24.2	17.7
Mg-5Sn-2Zn [22]		3%			233		14.1
		10%			260		10.6

**Table 2:** Influence of pre-cold deformation on peak aging time and precipitate size.

Materials	Pre-Deform	Pre-Strain	Peak Aging	Precipitates	Precipitate Size (nm)
As-cast	Forging (Multi-axial)	0%	200 °C/48 h	β″; β′	25 × 25 × 3; 25 × 25 × 3
WE43 [24]		15%	200 °C/32 h		15 × 15 × 2; 15 × 15 × 3 (Length × width × thickness)
Extruded	Rolling (ED)	0%	200 °C/36 h	β′	13.8 × 7.2
Mg-14Gd-0.5Zr alloy [38]		27%	200 °C/16 h		8.9 × 6.8 (Length × width)
Extruded	Tension (ED)	0%	150 °C/48 h	rod-like β′_1_	440 × 60
Mg–3Zn [55]		5%	150 °C/32 h		14 × 9 (Length × diameter)
Extruded	Tension (ED)	0%	150 °C/256 h	rod-like β′1	475 × 20
Mg-3Zn-0.5Y [52]		3%	150 °C/48 h		102 × 12
		5%	150 °C/32 h		67 × 10 (Length × diameter)
Rolled	Tension (RD)	0%	150 °C/48 h	Mg_2_Sn	204
Mg-5Sn-2Zn sheet [22]		3%	150 °C/48 h		149
		10%	150 °C/48 h		119 (Mean size)
As-cast	Rolling	0%	200 °C/36 h	β″	4.4 × 14.8
Mg-2.1Gd-1.7Ho-1.4Y-1.3Nd-0.9Er-0.5Zn-0.5Zr [26]		12%	200 °C/9 h		3.1 × 10.1 (Diameter × thickness)

## References

[1] Pan H., Ren Y., Fu H., Zhao H., Wang L., Meng X., Qin G. (2016). Recent developments in rare-earth free wrought magnesium alloys having high strength: A review. J. Alloy. Compd..

[2] Nie J.F. (2012). Precipitation and Hardening in Magnesium Alloys. Metall. Mater. Trans. A.

[3] Nie J. (2003). Effects of precipitate shape and orientation on dispersion strengthening in magnesium alloys. Scr. Mater..

[4] Robson J., Stanford N., Barnett M., Robson J. (2011). Effect of precipitate shape on slip and twinning in magnesium alloys. Acta Mater..

[5] Liu B.Y., Yang N., Wang J., Barnett M., Xin Y.C., Wu D., Xin R.L., Li B., Narayan R.L., Nie J.F. (2018). Insight from in situ microscopy into which precipitate morphology can enable high strength in magnesium alloys. J. Mater. Sci. Technol..

[6] Zhang M.X., Kelly P. (2003). Crystallography of Mg17Al12 precipitates in AZ91D alloy. Scr. Mater..

[7] Buha J. (2008). Reduced temperature (22–100°C) ageing of an MgZn alloy. Mater. Sci. Eng. A.

[8] Li T., Du Z., Zhang K., Li X., Yuan J., Li Y., Ma M., Shi G. (2012). Morphology and crystallography of β precipitate phase in Mg-Gd-Y-Nd-Zr alloy. Trans. Nonferr. Met. Soc. China.

[9] Xie H., Pan H., Ren Y., Sun S., Wang L., He Y., Qin G. (2018). Co-existences of the two types of β′ precipitations in peak-aged Mg-Gd binary alloy. J. Alloy. Compd..

[10] Kim S.H., Lee J.U., Kim Y.J., Bae J.H., You B.S., Park S.H. (2018). Accelerated precipitation behavior of cast Mg-Al-Zn alloy by grain refinement. J. Mater. Sci. Technol..

[11] Liu F., Xin R., Wang C., Song B., Liu Q. (2019). Regulating precipitate orientation in Mg-Al alloys by coupling twinning, aging and detwinning processes. Scr. Mater..

[12] Gao X., Nie J. (2007). Characterization of strengthening precipitate phases in a Mg–Zn alloy. Scr. Mater..

[13] Huang X., Han G., Huang W. (2018). T6 Treatment and Its Effects on Corrosion Properties of an Mg–4Sn–4Zn–2Al Alloy. Materials.

[14] Zhang R., Wang J., Huang S., Liu S., Pan F. (2017). Substitution of Ni for Zn on microstructure and mechanical properties of Mg–Gd–Y–Zn–Mn alloy. J. Magnes. Alloy..

[15] Tahreen N., Zhang D., Pan F., Jiang X., Li D., Chen D. (2018). Strengthening mechanisms in magnesium alloys containing ternary I, W and LPSO phases. J. Mater. Sci. Technol..

[16] Geng J., Gao X., Fang X., Nie J. (2011). Enhanced age-hardening response of Mg–Zn alloys via Co additions. Scr. Mater..

[17] Mendis C., Ohishi K., Kawamura Y., Honma T., Kamado S., Hono K. (2009). Precipitation-hardenable Mg–2.4Zn–0.1Ag–0.1Ca–0.16Zr (at.%) wrought magnesium alloy. Acta Mater..

[18] Muga C., Guo H., Xu S., Zhang Z., Zhang Z. (2017). Effects of aging and fast-cooling on the mechanical properties of Mg-14Li-3Al-3Ce alloy. Mater. Sci. Eng. A.

[19] Chen Y., Pan S., Tang S., Liu W., Tang C., Xu F. (2016). Formation mechanisms and evolution of precipitate-free zones at grain boundaries in an Al-Cu-Mg-Mn alloy during homogenization. J. Mater. Sci..

[20] Jiang J., Wu J., Ni S., Yan H., Song M. (2018). Improving the mechanical properties of a ZM61 magnesium alloy by pre-rolling and high strain rate rolling. Mater. Sci. Eng. A.

[21] Jia W., Ning F., Ding Y., Le Q., Tang Y., Cui J. (2018). Role of pre-width reduction in deformation behavior of AZ31B alloy during break-down rolling and finish rolling. Mater. Sci. Eng. A.

[22] Hu T., Xiao W., Wang F., Li Y., Lyu S., Zheng R., Ma C. (2018). Improving tensile properties of Mg-Sn-Zn magnesium alloy sheets using pre-tension and ageing treatment. J. Alloy. Compd..

[23] Song B., Yang Q., Zhou T., Chai L., Guo N., Liu T., Guo S., Xin R. (2019). Texture control by {10–12} twinning to improve the formability of Mg alloys: A review. J. Mater. Sci. Technol..

[24] Kang Y., Wang X., Zhang N., Yan H., Chen R. (2017). Effect of pre-deformation on microstructure and mechanical properties of WE43 magnesium alloy. Mater. Sci. Eng. A.

[25] Guo F., Zhang D., Fan X., Li J., Jiang L., Pan F. (2016). Microstructure, texture and mechanical properties evolution of pre-twinning Mg alloys sheets during large strain hot rolling. Mater. Sci. Eng. A.

[26] Li G., Zhang J., Wu R., Liu S., Song B., Jiao Y., Yang Q., Hou L. (2019). Improving age hardening response and mechanical properties of a new Mg-RE alloy via simple pre-cold rolling. J. Alloy. Compd..

[27] Song B., Wang C., Guo N., Pan H., Xin R. (2017). Improving Tensile and Compressive Properties of an Extruded AZ91 Rod by the Combined Use of Torsion Deformation and Aging Treatment. Materials.

[28] Wang C., Xin R., Li D., Song B., Wu M., Liu Q. (2017). Enhancing the age-hardening response of rolled AZ80 alloy by pre-twinning deformation. Mater. Sci. Eng. A.

[29] Wang C., Xin R., Li D., Song B., Liu Z., Liu Q. (2017). Tailoring the Microstructure and Mechanical Property of AZ80 Alloys by Multiple Twinning and Aging Precipitation. Adv. Eng. Mater..

[30] Yang P., Wang L.N., Xie Q.G., Li J.Z., Ding H., Lu L.L. (2011). Influence of deformation on precipitation in AZ80 magnesium alloy. Int. J. Miner. Metall. Mater..

[31] Pan H., Pan F., Peng J., Gou J., Tang A., Wu L., Dong H. (2013). High-conductivity binary Mg–Zn sheet processed by cold rolling and subsequent aging. J. Alloy. Compd..

[32] Chen X., Liu L., Pan F. (2016). Strength improvement in ZK60 magnesium alloy induced by pre-deformation and heat treatment. J. Wuhan Univ. Technol. Sci. Ed..

[33] Chen H., Liu T., Zhang Y., Song B., Hou D., Pan F. (2016). The yield asymmetry and precipitation behavior of pre-twinned ZK60 alloy. Mater. Sci. Eng. A.

[34] Song B., Xin R., Sun L., Chen G., Liu Q. (2013). Enhancing the strength of rolled ZK60 alloys via the combined use of twinning deformation and aging treatment. Mater. Sci. Eng. A.

[35] Zhao S., Guo E., Wang L., Wu T., Feng Y. (2015). Effect of pre-compressive strain on microstructure and mechanical properties of Mg–2.7Nd–0.4Zn–0.5Zr alloy. Mater. Sci. Eng. A.

[36] Zheng K., Dong J., Zeng X., Ding W., Ding W. (2008). Effect of pre-deformation on aging characteristics and mechanical properties of a Mg–Gd–Nd–Zr alloy. Mater. Sci. Eng. A.

[37] Li R., Xin R., Chapuis A., Liu Q., Fu G., Zong L., Yu Y., Guo B., Guo S. (2016). Effect of cold rolling on microstructure and mechanical property of extruded Mg–4Sm alloy during aging. Mater. Charact..

[38] Li R., Nie J., Huang G., Xin Y., Liu Q. (2011). Development of high-strength magnesium alloys via combined processes of extrusion, rolling and ageing. Scr. Mater..

[39] Kim Y.J., Kim S.H., Lee J.U., Choi J.O., Kim H.S., Kim Y.M., Kim Y., Park S.H. (2017). Effects of cold pre-forging on microstructure and tensile properties of extruded AZ80 alloy. Mater. Sci. Eng. A.

[40] Li S., Tang W., Chen R., Ke W. (2014). Effect of pre-induced twinning on microstructure and tensile ductility in GW92K magnesium alloy during multi-direction forging at decreasing temperature. J. Magnes. Alloy..

[41] Braszczyńska-Malik K. (2009). Discontinuous and continuous precipitation in magnesium—Aluminium type alloys. J. Alloy. Compd..

[42] Hong S.G., Park S.H., Lee C.S. (2010). Role of {10–12} twinning characteristics in the deformation behavior of a polycrystalline magnesium alloy. Acta Mater..

[43] Zhang Y., Liu T., Ding X., Xu S., He J., Chen H., Pan F., Lu L. (2014). The precipitation behavior of a pretwinned Mg–6Al–1Zn alloy and the effect on subsequent deformation. J. Mater. Res..

[44] Wang F., Hazeli K., Molodov K.D., Barrett C.D., Al-Samman T., Molodov D.A., Kontsos A., Ramesh K.T., el Kadiri H., Agnew S.R. (2018). Characteristic dislocation substructure in {10–12} twins in hexagonal metals. Scr. Mater..

[45] Sun Q., Zhang X., Shu Y., Tan L., Liu Q. (2016). Two types of basal stacking faults within {10–12} twin in deformed magnesium alloy. Mater. Lett..

[46] Sun Q., Zhang Q., Li B., Zhang X., Tan L., Liu Q. (2017). Non-dislocation-mediated basal stacking faults inside {10–11} twins. Scr. Mater..

[47] Liu Y., Jiang Q., Liu Y., Liu H., Lin T. (2018). Effect of twins on the crystallographic characteristics of the Mg 17 Al 12 phase in the pre-compressed AZ91 alloy. Mater. Lett..

[48] Gu X.F., Wang M., Shi Z.Z., Chen L., Yang P. (2018). Asymmetrical Precipitation on the {10–12} Twin Boundary in the Magnesium Alloy. Metall. Mater. Trans. A.

[49] Yan K., Bai J., Liu H., Jin Z.Y. (2017). The precipitation behavior of MgZn 2 and Mg 4 Zn 7 phase in Mg-6Zn (wt.%) alloy during equal-channel angular pressing. J. Magnes. Alloy..

[50] Shi G.L., Zhang D.F., Zhang H.J., Zhao X.B., Qi F.G., Zhang K. (2013). Influence of pre-deformation on age-hardening response and mechanical properties of extruded Mg–6%Zn–1%Mn alloy. Trans. Nonferr. Met. Soc. China.

[51] Li Q., Huang G., Huang X., Pan S., Tan C., Liu Q. (2017). On the texture evolution of Mg–Zn–Ca alloy with different hot rolling paths. J. Magnes. Alloy..

[52] Rosalie J.M., Somekawa H., Singh A., Mukai T. (2013). Effect of precipitation on strength and ductility in a Mg–Zn–Y alloy. J. Alloy. Compd..

[53] Singh A., Tsai A.P. (2007). Structural characteristics of β_1_ precipitates in Mg-Zn-based alloys. Scr. Mater..

[54] Clark J. (1965). Transmission electron microscopy study of age hardening in a Mg-5 wt.% Zn alloy. Acta Met..

[55] Rosalie J.M., Somekawa H., Singh A., Mukai T. (2012). The effect of size and distribution of rod-shaped β_1_’ precipitates on the strength and ductility of a Mg-Zn alloy. Mater. Sci. Eng. A.

[56] Hadadzadeh A., Wells M.A. (2017). Analysis of the hot deformation of ZK60 magnesium alloy. J. Magnes. Alloy..

[57] Chen H., Kang S.B., Yu H., Cho J., Kim H.W., Min G. (2009). Effect of heat treatment on microstructure and mechanical properties of twin roll cast and sequential warm rolled ZK60 alloy sheets. J. Alloy. Compd..

[58] Song B., Xin R., Guo N., Xu J., Sun L., Liu Q. (2015). Dependence of tensile and compressive deformation behavior on aging precipitation in rolled ZK60 alloys. Mater. Sci. Eng. A.

[59] Liu Z., Xin R., Wu X., Liu D., Liu Q. (2018). Improvement in the strength of friction-stir-welded ZK60 alloys via post-weld compression and aging treatment. Mater. Sci. Eng. A.

[60] Jiang M., Xu C., Nakata T., Yan H., Chen R., Kamado S. (2017). Enhancing strength and ductility of Mg-Zn-Gd alloy via slow-speed extrusion combined with pre-forging. J. Alloy. Compd..

[61] Shao X., Peng Z., Jin Q., Ma X. (2017). Atomic scale characterizing interaction between {10–13} twin and stacking faults with solute atoms in an Mg-Zn-Y alloy. Mater. Sci. Eng. A.

[62] Li Z., Wan D., Huang Y., Ye S., Hu Y. (2017). Characterization of a Mg95.5Zn1.5Y3 alloy both containing W phase and LPSO phase with or without heat treatment. J. Magnes. Alloy..

[63] Singh A., Osawa Y., Somekawa H., Mukai T. (2018). Effect of Solidification Cooling Rate on Microstructure and Mechanical Properties of an Extruded Mg-Zn-Y Alloy. Metals.

[64] Zhu W., Liu W., Zhang Y.G., Ma Q.Q., Zhang J., Xu C. (2019). Influence of Ni Alloying on the Precipitation of Quasicrystal Phase in As-Cast Mg 96.5 Zn 1 Y 1.5 Mn 1 Alloy. Adv. Eng. Mater..

[65] Ye J., Lin X.P., Zhao T.B., Liu N.N., Xie H.B., Niu Y., Teng F. (2016). Influence of pre-strain on the aging hardening effect of the Mg-9.02Zn-1.68Y alloy. Mater. Sci. Eng. A.

[66] Ren W., Xin R., Xu J., Song B., Zhang L., Liu Q. (2019). Effects of precipitate type on twin/slip activity in ZK60 alloys and yield asymmetry. J. Alloy. Compd..

[67] Li Z., Zhang J., Feng Y., Xie J., Liu Y., Liu S., Meng J., Yang Q., Liu Z., Wu R. (2019). Development of Hot-Extruded Mg–RE–Zn Alloy Bar with High Mechanical Properties. Materials.

[68] Jung I.H., Sanjari M., Kim J., Yue S. (2015). Role of RE in the deformation and recrystallization of Mg alloy and a new alloy design concept for Mg–RE alloys. Scr. Mater..

[69] Majd A.M., Farzinfar M., Pashakhanlou M., Nayyeri M.J. (2018). Effect of RE elements on the microstructural and mechanical properties of as-cast and age hardening processed Mg–4Al–2Sn alloy. J. Magnes. Alloy..

[70] Gao Z., Hu L., Li J., An Z., Li J., Huang Q. (2018). Achieving High Strength and Good Ductility in As-Extruded Mg–Gd–Y–Zn Alloys by Ce Micro-Alloying. Materials.

[71] You S., Huang Y., Kainer K.U., Hort N. (2017). Recent research and developments on wrought magnesium alloys. J. Magnes. Alloy..

[72] Zhang J., Liu S., Wu R., Hou L., Zhang M. (2018). Recent developments in high-strength Mg-RE-based alloys: Focusing on Mg-Gd and Mg-Y systems. J. Magnes. Alloy..

[73] Liu H., Gao Y., Liu J., Zhu Y., Wang Y., Nie J. (2013). A simulation study of the shape of β’ precipitates in Mg–Y and Mg–Gd alloys. Acta Mater..

[74] Xin R., Li L., Zeng K., Song B., Liu Q. (2011). Structural examination of aging precipitation in a Mg–Y–Nd alloy at different temperatures. Mater. Charact..

[75] Hilditch T., Nie J., Muddle B.C., Mordike B.L., Kainer K.U. (1998). Werkstoff Informationsgesellschaft. Magnesium Alloys and Their Applications.

[76] Čížek J., Procházka I., Smola B., Stulíková I., Očenášek V. (2007). Influence of deformation on precipitation process in Mg–15wt.%Gd alloy. J. Alloy. Compd..

[77] Fang D., Ma N., Cai K., Cai X., Chai Y., Peng Q. (2014). Age hardening behaviors, mechanical and corrosion properties of deformed Mg–Mn–Sn sheets by pre-rolled treatment. Mater. Des..

[78] Peng Q., Ma N., Li X., Zhang J. (2012). Age hardening response of a Mg–5wt.%Sc alloy under an applied stress field. Mater. Lett..

[79] Zhao H., Pan J., Li H., Tian Y., Peng Q. (2013). Spherical strengthening precipitate in a Mg-10wt%Y alloy with superhigh pressure aging. Mater. Lett..

[80] Agnew S.R., Duygulu O. (2005). Plastic anisotropy and the role of non-basal slip in magnesium alloy AZ31B. Int. J. Plast..

[81] Geng J., Chun Y., Stanford N., Davies C., Nie J., Barnett M. (2011). Processing and properties of Mg–6Gd–1Zn–0.6Zr. Mater. Sci. Eng. A.

[82] Jain J., Cizek P., Poole W.J., Barnett M.R. (2015). The role of back stress caused by precipitates on {10–12} twinning in a Mg–6Zn alloy. Mater. Sci. Eng. A.

[83] Song B., Pan H., Chai L., Guo N., Zhao H., Xin R. (2017). Evolution of gradient microstructure in an extruded AZ31 rod during torsion and annealing and its effects on mechanical properties. Mater. Sci. Eng. A.

[84] Zhang W., Huo W., Lu J., Hu J., Wei Q., Zhang Y. (2017). Gradient shear banding in a magnesium alloy induced by sliding friction treatment. Vacuum.

[85] Lu K. (2014). Making strong nanomaterials ductile with gradients. Science.

[86] Wu X., Zhu Y. (2017). Heterogeneous materials: A new class of materials with unprecedented mechanical properties. Mater. Res. Lett..

[87] Luo X., Huang T., Wang Y., Xin Y., Wu G. (2019). Strong and ductile AZ31 Mg alloy with a layered bimodal structure. Sci. Rep..

[88] He J., Jin L., Wang F., Dong S., Dong J. (2017). Mechanical properties of Mg-8Gd-3Y-0.5Zr alloy with bimodal grain size distributions. J. Magnes. Alloy..

[89] Pan H., Fu H., Song B., Ren Y., Zhao C., Qin G. (2016). Formation of profuse dislocations in deformed calcium-containing magnesium alloys. Philos. Mag. Lett..

[90] Pan H., Huang Q., Qin G., Fu H., Xu M., Ren Y., She J., Song B., Li B. (2017). Activations of stacking faults in the calcium-containing magnesium alloys under compression. J. Alloy. Compd..

[91] Wang F., Agnew S.R. (2016). Dislocation transmutation by tension twinning in magnesium alloy AZ31. Int. J. Plast..

[92] Wang L., Song B., Zhang Z., Zhang H., Han T., Cao X., Wang H., Cheng W. (2018). Enhanced Stretch Formability of AZ31 Magnesium Alloy Thin Sheet by Induced Precompression and Sequent Annealing. Materials.

[93] Bailey J.E., Hirsch P.B. (1960). The dislocation distribution, flow stress, and stored energy in cold-worked polycrystalline silver. Philos. Mag..

[94] Song B., Guo N., Liu T., Yang Q. (2014). Improvement of formability and mechanical properties of magnesium alloys via pre-twinning: A review. Mater. Des..

[95] Wen H., Topping T.D., Isheim D., Seidman D.N., Lavernia E.J. (2013). Strengthening mechanisms in a high-strength bulk nanostructured Cu–Zn–Al alloy processed via cryomilling and spark plasma sintering. Acta Mater..

[96] Zhu S., Ringer S.P. (2018). On the role of twinning and stacking faults on the crystal plasticity and grain refinement in magnesium alloys. Acta Mater..

[97] Wang F., Bhattacharyya J.J., Agnew S.R. (2016). Effect of precipitate shape and orientation on Orowan strengthening of non-basal slip modes in hexagonal crystals, application to magnesium alloys. Mater. Sci. Eng. A.

[98] Xu C., Nakata T., Qiao X.G., Zheng M.Y., Wu K., Kamado S. (2017). Ageing behavior of extruded Mg–8.2Gd–3.8Y–1.0Zn–0.4Zr (wt.%) alloy containing LPSO phase and γ’ precipitates. Sci. Rep..

[99] Zhang X., Li B., Liu Q. (2015). Non-equilibrium basal stacking faults in hexagonal close-packed metals. Acta Mater..

[100] Li B., Yan P., Sui M., Ma E., Yan P. (2010). Transmission electron microscopy study of stacking faults and their interaction with pyramidal dislocations in deformed Mg. Acta Mater..

[101] Sandlöbes S., Friák M., Zaefferer S., Dick A., Yi S., Letzig D., Pei Z., Zhu L.F., Neugebauer J., Raabe D. (2012). The relation between ductility and stacking fault energies in Mg and Mg–Y alloys. Acta Mater..

[102] Song B., Shu X., Pan H., Li G., Guo N., Liu T., Chai L., Xin R. (2017). Influence of Torsion Route on the Microstructure and Mechanical Properties of Extruded AZ31 Rods. Adv. Eng. Mater..

[103] Song B., Liu T., Xin R., Yang H., Guo N., Chai L., Huang Y., Hort N. (2019). Influence of Torsion on Precipitation and Hardening Effects during Aging of an Extruded AZ91 Alloy. J. Mater. Eng. Perform..

